# Supplementary material for: Splice-Junction-Based Mapping of Alternative Isoforms in the Human Proteome
Source: Cell Rep. Author manuscript; Available in PMC 2020 Jan 15. (PMC6961840; doi:10.1016/j.celrep.2019.11.026)
Supplement: 2 [file NIHMS1546469-supplement-2.zip › DF1/PXD010154/Lung/Lung_30_SRSF1_ALGLVGRPVLESALFVTAMSGGGVIR.pdf]

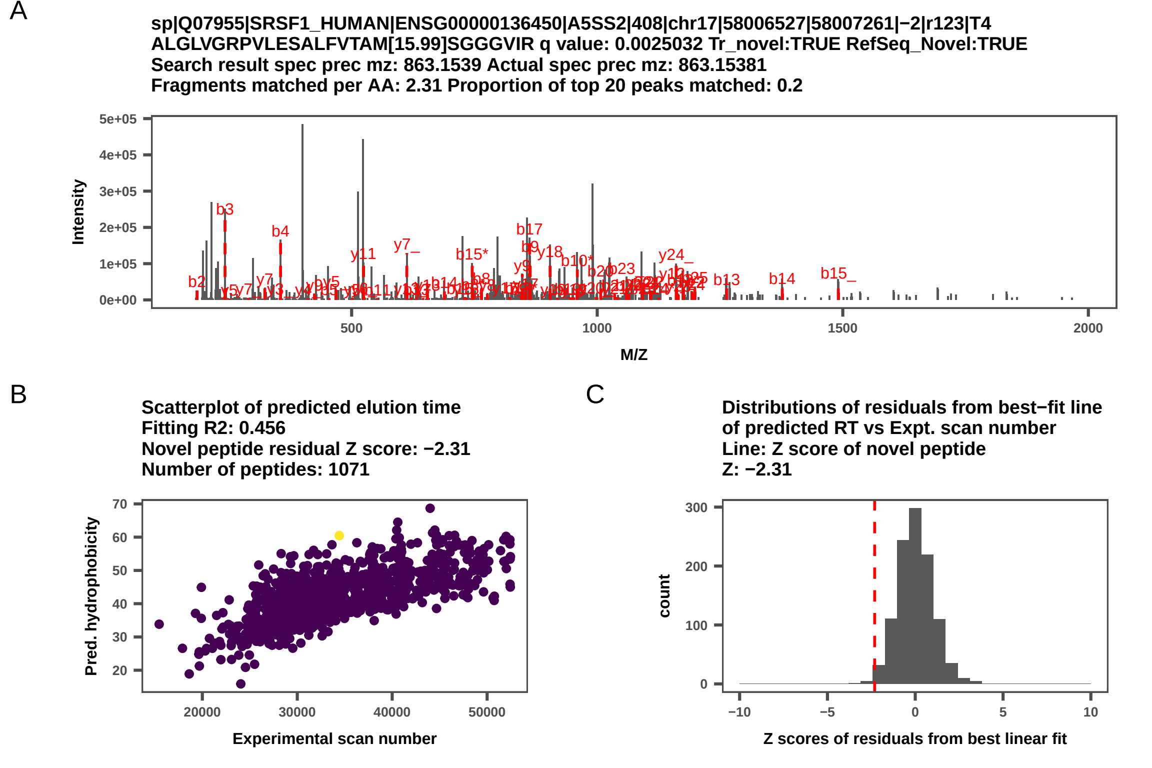
A
sp|Q07955|SRSF1_HUMAN|ENSG00000136450|A5SS2|408|chr17|58006527|58007261|−2|r123|T4
ALGLVGRPVLESALFVTAM[15.99]SGGGVIR q value: 0.0025032 Tr_novel:TRUE RefSeq_Novel:TRUE
Search result spec prec mz: 863.1539 Actual spec prec mz: 863.15381
Fragments matched per AA: 2.31 Proportion of top 20 peaks matched: 0.2
Intensity
5e+05
4e+05
3e+05
2e+05
1e+05
0e+00
b3
b4
b2
y11
y7_
b15*
b17
b9
y18
y9
b10*
b20
b23
y24_
b13
b14
b15_
500
1000
1500
2000
M/Z
B
Scatterplot of predicted elution time
Fitting R2: 0.456
Novel peptide residual Z score: −2.31
Number of peptides: 1071
Pred. hydrophobicity
70
60
50
40
30
20
20000
30000
40000
50000
Experimental scan number
C
Distributions of residuals from best−fit line
of predicted RT vs Expt. scan number
Line: Z score of novel peptide
Z: −2.31
count
300
200
100
0
−10
−5
0
5
10
Z scores of residuals from best linear fit
